# Polarclean as a Sustainable Reaction Medium for the Waste Minimized Synthesis of Heterocyclic Compounds

**DOI:** 10.3389/fchem.2018.00659

**Published:** 2019-01-29

**Authors:** Francesco Ferlin, Lorenzo Luciani, Orlando Viteritti, Francesco Brunori, Oriana Piermatti, Stefano Santoro, Luigi Vaccaro

**Affiliations:** Laboratory of Green Synthetic Organic Chemistry, Dipartimento di Chimica, Biologia e Biotecnologie, Università di Perugia, Perugia, Italy

**Keywords:** heterocycles, green-synthesis, isoxazoles, triazoles, C–H activation

## Abstract

Herein we report the use of Rhodiasolv^©^ Polarclean as a novel polar aprotic solvent for the synthesis of decorated heterocycles via dipolar cycloaddition (isooxazoles) or intramolecular C–H functionalization processes (benzo-fused chromenes). The use of Polarclean allowed to isolate the final products in good yields by simple solid filtration or liquid-liquid phase separation, avoiding the need for chromatographic purification. Moreover, since in the synthesis of benzo-fused chromenes, the metal catalyst is retained in Polarclean, the catalyst/reaction medium can be easily reused for consecutive reaction runs, without any apparent loss in efficiency. This methodology is associated with a limited waste production. These results extend the applicability of Polarclean as a promising reaction medium for the replacement of toxic petrol-based solvent.

## Introduction

Heterocyclic compounds are ubiquitous and find multiple applications in different fields of applied chemistry such as in medicinal chemistry, as key motifs in pharmaceutically active ingredients (Gomtsyan, 2012), and in material science (Yin and Shreeve, 2017). Thus, chemists have always been looking for novel synthetic methodologies that would allow to access heterocyclic cores in more efficient, economical and selective ways and, as a result, many efficient examples are available in the literature. The most effective and straightforward way to access heterocyclic cores is probably still represented by cycloaddition reactions (Heravi et al., 2015; Padwa and Bur, 2016). These reactions typically occur with perfect atom economy and, since they allow the simultaneous formations of two bonds, they are generally also very efficient in terms of step economy. One of the possible limitations of cycloadditions reactions is that often they have rather strict structural requirements on the substrates for the cycloaddition to occur, which results in the potential need for subsequent transformations to decorate the heterocyclic core and access the target molecule.

In recent years, great advancements in transition metals catalyzed reactions provided synthetic organic chemists with many more tools to efficiently obtain heterocyclic molecules (Gulevich et al., 2013). In particular, the last decade saw enormous improvements in the available methodologies to activate and directly functionalize C–H bonds, and many of these methodologies indeed are specifically directed toward the synthesis of heterocycles (Thansandote and Lautens, 2009; Mei et al., 2012; Inamoto, 2013). However, these reactions typically require hazardous conditions and the use of common toxic organic solvents and suffer from procedural limitations such as the need of strictly anhydrous conditions. The need to develop more sustainable procedures for chemical production has recently brought some results also in the realm of C–H functionalization methodologies, particularly in the use of recoverable and reusable catalysts (Santoro et al., 2016) and of benign bio-based reaction media (Santoro et al., 2017, 2018). In fact, waste disposal represents one of the major issues related to chemical productions due to economic and environmental reasons. Increasingly stringent regulations impose strict limitations on the use of toxic organic solvents and more in general to the large use of potentially harmful substances and volatile organic compounds. Solvents constitute the largest portion of the waste associated to a chemical process and the prime responsible for the related CO_2_ emissions (Bruntland's report the World Commission on Environmental Development, 1987; Pollution Prevention Act, 1990; Anastas and Warner, 1998; Jiménez-González et al., 2004; Jimenez-Gonzalez et al., 2011).

As a part of our research program devoted to the search for novel environmentally benign reaction media, we are interested in the use of sustainable green solvents in the synthesis and functionalization of heterocycle systems (Rasina et al., 2016; Tian et al., 2016; Ferlin et al., 2017, 2018a; Bechtoldt et al., 2018; Vaccaro et al., 2018). In this context, we have recently reported the use of Rhodiasolv^©^ Polarclean as an efficient system for the waste-minimized synthesis of fully decorated 1,2,3-triazoles (Luciani et al., 2018). Rhodiasolv^©^ Polarclean, is composed by methyl-5-(dimethylamino)-2-methyl-5-oxopentanoate and its diamide derivative in a 20:1 ratio. It is commercially available and finds application as a solvent, co-solvent, or crystal growth inhibitor in agrochemical formulations (Vidal, 2012). It is miscible with water and has a boiling point of 278–282°C and a melting point of −60°C. Polarclean is industrially produced from methyleneglutarodinitrile (MDN), a by-product of Nylon-66 manufacturing, otherwise needed to be burnt to be disposed (Vidal, 2012). To the best of our knowledge Polarclean has been rarely used as a reaction medium and it has been tested among other solvents in metathesis polymerization (Lebarbé et al., 2014), olefin epoxidation (Mouret et al., 2014), and fiber membranes fabrication (Hassankiadeh et al., 2015).

In this contribution, we report our results on the use of Polarclean for the synthesis of widely interesting heterocyclic such as isoxazoles and polycyclic fused 1,2,3-triazoles. These heterocyclic systems are rather common and, for instance, triazole moiety is present in active pharmaceutical ingredients (Wu et al., 2018) as well as in optoelectronics and material sciences (Marrocchi et al., 2016). Isoxazoles are recognized as privilege structures for the synthesis of beta-lactamase resistant antibiotics (Decuyper et al., 2018), and recently they found application in the field of lithium ion batteries (Yang et al., 2017).

**Figure 1:**
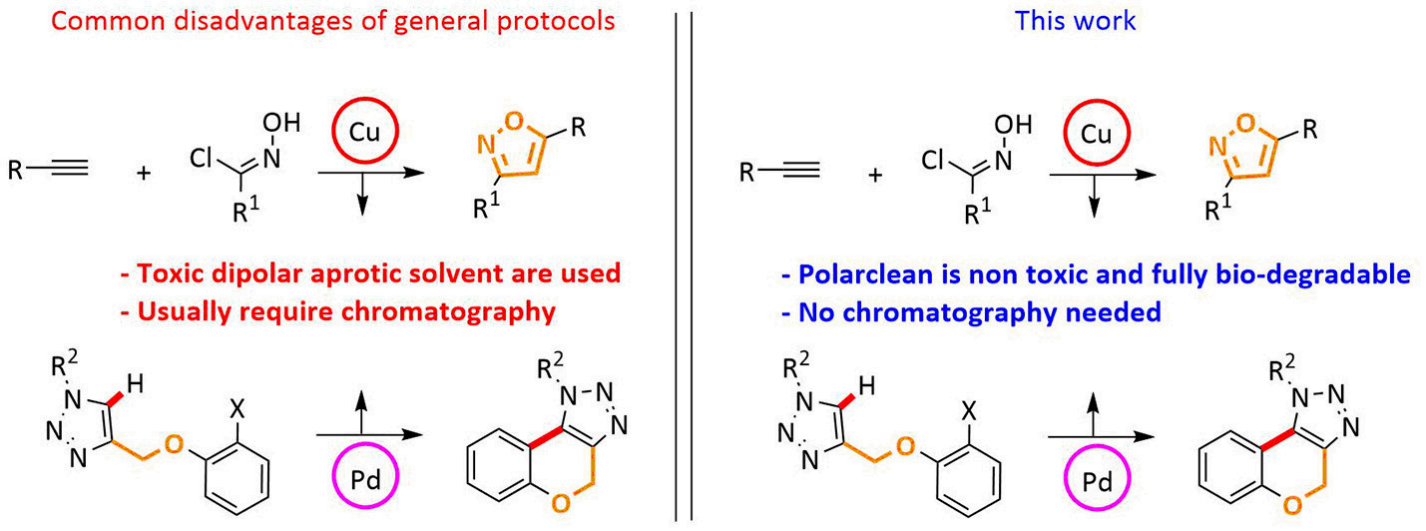
Features of current work.

Our investigations were directed toward the definition of protocols featuring recycle and reuse of solvent/catalyst systems, avoidance of wasteful chromatographic purification, and therefore minimization of waste production (Figure 1).

## Results and Discussion

We started our investigation by testing the use of Polarclean in the representative reaction of phenylacetylene (**1a**) with 4-bromo-*N*-hydroxybenzimidoyl chloride (**2a**), using 2 mol% of CuSO_4_·5H_2_O as copper source together with 10 mol% of sodium ascorbate as a reductant (Himo et al., 2005) (Table 1). The reaction was tested at 70°C for 24 h in Polarclean 1 M as medium and the corresponding isoxazole **3a** was obtained in 40% yield (Table 1, entry 1). In this case, due to high solubility of **3a** in pure Polarclean, the pure product could only be isolated after a classic purification procedure (aqueous work-up followed by column chromatography). Slightly better results were achieved when a 9:1 mixture of Polarclean/water was used as medium at 70°C (Table 1, entry 2). In these conditions, the reaction mixture was partially heterogeneous and product **3a** precipitated while forming and could be isolated in 50% yield by simple filtration. Increasing the amount of water by using a 4:1 Polarclean/H_2_O mixture lead to a further improvement in reaction yield, which reached 60% (Table 1, entry 3). An attempt to further increase the amount of water relative to the substrates and product while keeping the 4:1 Polarclean/H_2_O ratio, thus performing the reaction at 0.5 M concentration, resulted in drastically lower yield (33%, Table 1, entry 4). Finally, optimal results were obtained when Polarclean and water were used in 4:1 ratio at 1 M concentration and at 50°C (Table 1, entry 5). In these conditions in fact pure product **3a** could be obtained in 70% yield by simple filtration as it precipitates in the reaction mixture. The beneficial effect of lowering the temperature can possibly be attributed to a reduced degradation of imidoyl chloride **2a**.

**Table 1:** Optimization of reaction conditions for the synthesis of **3a**^**a**^.

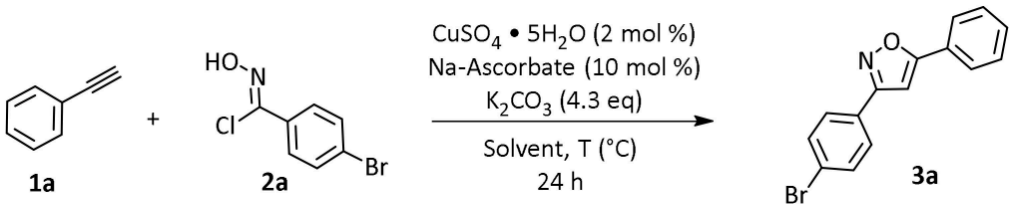			
**Entry**	**Solvent**	**T (°C)**	**Yield (%)**^**b**^
1	Polarclean 1 M	70	40
2	Polarclean/H_2_O (9:1) 1 M	70	50
3	Polarclean/H_2_O (4:1) 1 M	70	60
4	Polarclean/H_2_O (4:1) 0.5 M	70	33
5	Polarclean/H_2_O (4:1) 1 M	50	70

a *Reaction conditions: **1a** (1 mmol), **2a** (1 mmol), CuSO_4_ pentahydrate (2 mol %), Na-Ascorbate (10 mol %), K_2_CO_3_ (4.3 equivalent)*.

b *Isolated yield of **3a***.

**Scheme 1:**
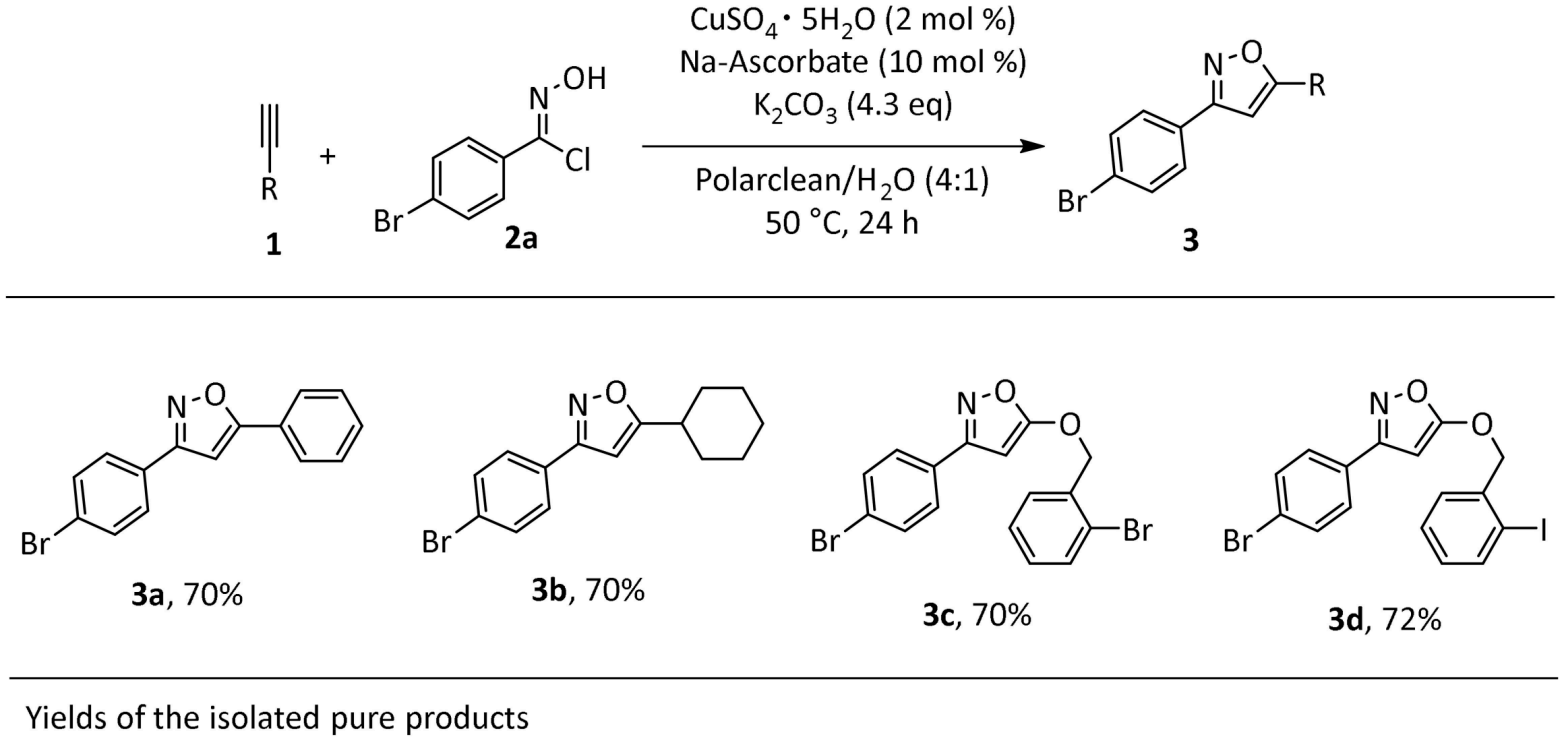
Scope of isoxazole **3a-d**^a^. ^*a*^Reaction conditions: **1a** (1 mmol), **2a** (1 mmol), CuSO_4_ pentahydrate (2 mol%), Na-Ascorbate (10 mol %), K_2_CO_3_ (4.3 equivalent), Polarclean/H_2_O (4:1) 1 M, 1mL.

The identified optimal reaction conditions were then applied to investigate the substrate scope. The protocol worked smoothly using combinations of aryl- or alkyl-substituted alkynes in combination with imidoyl chloride **2a**, affording the products in good yields (Scheme 1). Importantly, the presence of halogen substituents on the aromatic rings was well-tolerated, potentially allowing for late stage transformations of these functionalities. Very importantly, the final work-up for the synthesized products was consistent with our initial intent. In fact, the products were insoluble in the reaction media and in all cases precipitated at the end of the reaction, thus allowing a very easy isolation by filtration and washing with water to remove solvent impurities.

Next, we began our investigation on the use of Polarclean as reaction medium in the cyclization reaction by C–H functionalization of 1,2,3-triazole **4a** (Table 2). Starting from our experience in this transformation (Ferlin et al., 2018b), which suggested the use of simple Pd(OAc)_2_ as catalyst with a substoichiometric amount of 2,4,6-trimethylbenzoic acid as additive and potassium carbonate as base, we performed the reaction using Polarclean, pure or in combination with different amount of water, as reaction medium (Table 2).

**Table 2:** Optimization of reaction conditions for the cyclization of **4a** to **5a**^a^.

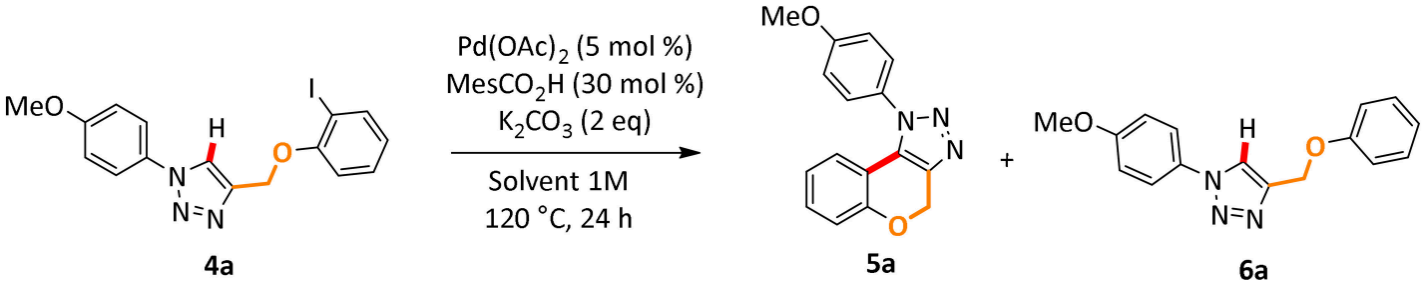			
**Entry**	**Solvent**	**Selectivity**^**b**^ **5a:6a**	**Yield of 5a (%)**^**c**^
1	Polarclean/H_2_O (4:1) 1 M	2:88	–
2	Polarclean/H_2_O (9:1) 1 M	5:85	–
3	Polarclean 1 M	99:1	87%

a *Reaction conditions: **4a** (1 mmol), MesCO_2_H (30 mol %, 0.3 mmol), K_2_CO_3_ (2 equivalent, 2 mmol), Pd(OAc)_2_ (5 mol %, 0.05 mmol)*.

b *Measured by GC analyses using samples of pure compounds as reference*.

c *Isolated yield*.

**Scheme 2:**
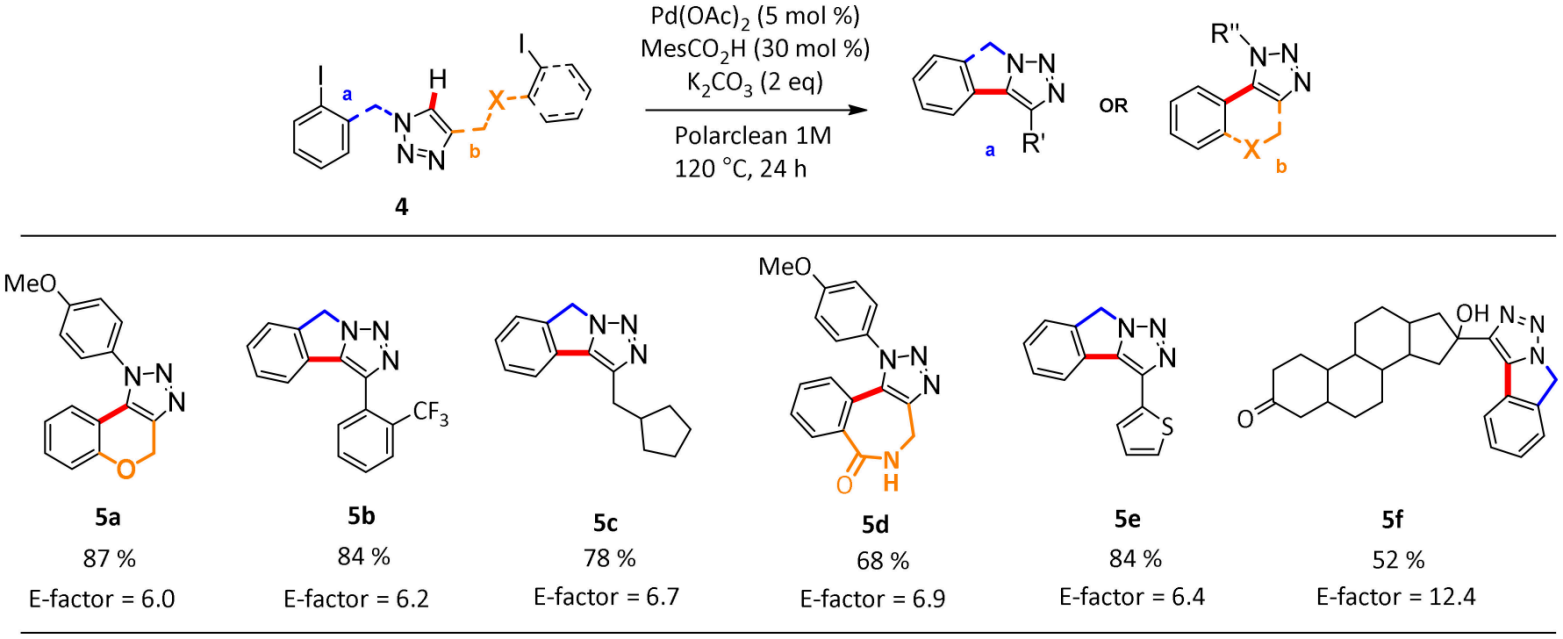
Scope of hetero-fused triazoles **5a-f**^a^. ^*a*^Data reported refer to the isolated yield of the pure product. ^*a*^Reaction conditions: **4a** (1 mmol), MesCO_2_H (30 mol %, 0.3 mmol), K_2_CO_3_ (2 equivalent, 2 mmol), Pd(OAc)_2_ (5 mol %, 0.05 mmol), Polarclean 1 mL, 1 M.

In this process, the best selectivity was found when pure Polarclean was used as reaction medium (Table 2, entry 3). In fact, the presence of water influenced dramatically the final composition of the reaction mixture, favoring the formation of the de-halogenated side-product **6a** (Table 2, entries 1 and 2). With the optimized reaction conditions, we further explored the scope of this process (Scheme 2). A wide range of substrates could be employed in the intramolecular C–H arylation of 1,2-3-triazole-based substrates **4**, giving access to either triazolo-fused chromenes or triazolo-fused isoindoles, depending on the substitution pattern on the triazole substrate. The reaction is compatible with the presence of an oxygen atom in the side-chain, giving rapid access to benzo-fused chromene **5a** in 87% yield. Aryl- or alkyl-substituted [1,2,3]-triazolo[5,1-a]isoindoles **5b**, **5c**, and **5e** could also be obtained in good yields (78–84%). To our delight, the optimized reaction conditions proved effective on more complex substrates, giving access both to triazolo-fused benzazepine **5d** and to the steroid-substituted [1,2,3]-triazolo[5,1-a]isoindoles **5f** in 68 and 52% yields, respectively. In some cases (**5b**, **5d**, and **5f**) the formation of traces amount of dehalogenation products (<2%) was observed.

Also in this case the isolation of the product was conducted *via* re-crystallization without the need for further purification, except in the case of the steroid product **5f** in which filtration over a silica pad and precipitation in water were necessary to achieve the pure compound. Finally, we also investigated the recycle and reuse of the solvent/catalyst system (Table 3).

**Table 3:** Recycle of solvent/catalyst system for the synthesis of representative compound **5a**^a^.

	**1st run**	**2nd run**	**3rd run**
Selectivity **5a:6a**^b^	99:1	95:5	92:8
Yield of **5a** (%)^c^	87%	82%	78%

a *Reaction conditions: **4a** (1 mmol), MesCO_2_H (30 mol %, 0.3 mmol), K_2_CO_3_ (2 equivalent, 2 mmol), Pd(OAc)_2_ (5 mol %, 0.05 mmol)*.

b *Measured by GC analyses using samples of pure compounds as reference*.

c *Isolated yield of the pure **5a***.

We found that for almost all of the substrates it was possible to filtrate the reaction mixture on a Büchner funnel, collecting the product, and reuse the solvent system, which also retains the palladium catalyst, without any treatment for at least three consecutive cycles and with a limited loss in efficiency and selectivity (Table 3). The latter is likely caused by an increase in water content of the solvent/catalyst system over consecutive reaction runs, which was already demonstrated to be detrimental for the selectivity of the process.

We also calculated the green metrics associated with the C–H functionalization protocol to compare the results of the reactions conducted in Polarclean with those obtained using other media. We were pleased to find that, compared to other common synthetic protocols present in literature (see Supplementary Information), the use of our recyclable system for the intramolecular C–H activation allows us to achieve very low E-factor values around 6 for the synthesis of polycyclic heterocycles (Scheme 2). The only exception is represented by the cyclization of the steroid substituted substrate to give product **5f**, for which recycling of the solvent/catalyst system was hampered by the necessity to add water to isolate the pure product.

## Conclusion

In conclusion, we have reported that Polarclean, a novel solvent deriving from the waste valorization of Nylon 66 manufacturing, can be an effective alternative to common petrol-based solvents in the reactions object of the current investigation. Dipolar cycloadditions benefit from the use of Polarclean in terms of isolation of final products and therefore in achieving a waste minimized protocol for the synthesis of isoxazole **3**. Intramolecular C–H activation also proved to be feasible using Polarclean allowing the synthesis of polycyclic heterocycles **5** in a step and atom economical fashion and with the reuse of the medium/catalyst system, thus effectively minimizing the waste generation.

## Author Contributions

FF, LL, OV, FB, OP, and SS performed the experiments. FF and LV contributed to conception and design of the study. SS and LV wrote the manuscript. All authors contributed to manuscript revision, read and approved the submitted version.

### Conflict of Interest Statement

The authors declare that the research was conducted in the absence of any commercial or financial relationships that could be construed as a potential conflict of interest.
