# Supplementary material for: Polarclean as a Sustainable Reaction Medium for the Waste Minimized Synthesis of Heterocyclic Compounds
Source: Front Chem. 2019 Jan 29;6:659. doi: 10.3389/fchem.2018.00659 (PMC6362304; doi:10.3389/fchem.2018.00659)
Supplement: Supplementary file 1 [file Data_Sheet_1.PDF]

Supplementary Material

Article Title

Francesco Ferlin, Lorenzo Luciani, Orlando Viteritti, Francesco Brunori, Oriana Piermatti, Stefano Santoro, Luigi Vaccaro

Laboratory of Green Synthetic Organic Chemistry – Dipartimento di Chimica, Biologia e Biotecnologie, Università di Perugia Via Elce di Sotto, 8 - 06123 Perugia, Italy

*** Correspondence:** Luigi Vaccaro, luigi.vaccaro@unipg.it

General informations:

GC analyses were performed using a Hewlett- Packard HP 5890A equipped with a capillary column DB35MS (30 m, 0.53 mm), a FID detector and helium as a gas carrier. GC-EIMS analyses were carried out using a Hewlett-Packard HP 6890N Network GC system/5975 Mass Selective Detector equipped with an electron impact ionizer at 70 eV. NMR spectra were recorded on a Bruker DRX-ADVANCE 400 MHz (1H at 400 MHz and 13C at 100.6 MHz and 19F at 376 MHz) in $CDCl_3$ or DMSO-d_6. Elemental analyses were conducted on a FISONS instrument EA 1108 CHN. Melting points were measured on a Büchi 510 instrument. Unless otherwise stated, all solvents and reagents were purchased and used without further purification.

General Procedures 1 for the CuAAC reaction in Polarclean: synthesis of isoxazoles 3a-d

in a screw-capped vial equipped with a magnetic stirring bar the reagents were charged in the following order: imidoyl chloride (**2a**, 1 mmol, 1 eq), alkyne (1 mmol, 1 eq), copper sulfate pentahydrate (0.02 mmol, 0.02 eq.) sodium ascorbate (0.1 mmol, 0.1 eq.), and K_2CO_3 (4.3 mmol, 4.3 eq). 2 mL of Polarclean/water (4:1) was added and the reaction was heated to 50 °C under stirring for 24 h. In all the cases the formation of the product is evidenced by the formation of a precipitate. The product was filtered with a Büchner funnel, washed with water (1 mL) and dried under vacuum.

General Procedures 2 for the intramolecular C–H activation in Polarclean: synthesis of compound 5a-f

in a screw-capped vial equipped with a magnetic stirring bar the reagents were charged in the following order: triazole (1 mmol, 1 eq), $MesCO_2H$ (0.3 mmol, 0.3 eq), K_2CO_3 (2 mmol, 2 eq), $Pd(OAc)_2$ (0.05 mmol, 0.05 eq). 1 mL of pure Polarclean was added and the reaction was heated to 120 °C under stirring for 24 h. In most of the case the formation of the product is evidenced by the formation of a precipitate. The product was filtered with a Büchner funnel, washed with water (1 mL) and dried under vacuum.

Characterization data for compound 3a-d

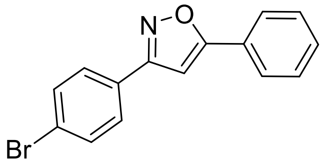

3-(4-bromophenyl)-5-phenylisoxazole (3a):

Compound **3a** was synthesized in 70 % yield using general procedure 1. M.p. 182-184 °C. ^{1}H NMR (Methanol-d_4) : 7.96 – 7.92 (m, 4H), 7.75 (d, J = 8 Hz, 2H), 7.61 – 7.55 (m, 3H), 7.43 (s, 1H). ^{13}C NMR : 132.2, 131.8, 130.4, 129.2, 128.5, 128.1, 127.4, 126.1, 125.7, 123.8. GC-EIMS (m/z): 301 (17), 299 (18), 105 (100), 77 (53). Elemental Analysis for $C_{15}H_{10}BrNO$: calcd C, 60.02; H, 3.36; N, 4.67; found C, 60.08; H 3.24; N, 4.58.

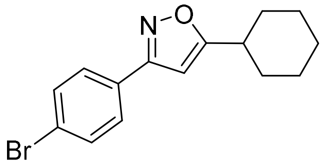

3-(4-bromophenyl)-5-cyclohexylisoxazole 3b:

Compound **3b** was synthesized in 70 % yield using general procedure 1. M.p. 168-169 °C. ^{1}H NMR (Methanol-d_4) : 7.80 (d, J = 8 Hz, 2H), 7.71 (d, J = 12 Hz, 2H), 6.81 (s, 1H), 2.84 (s, 1H), 2.01 (d, J = 8 Hz, 2H), 1.76 (d, J = 12 Hz, 2H), 1.67 (d, J = 16 Hz, 1H), 1.47 – 1.23 (m, 5H). ^{13}C NMR :178.8, 161.2, 129.0, 128.6, 124.0, 98.2, 36.0, 25.8, 25.5. GC-EIMS (m/z): 307 (44), 305 (45), 224 (99), 222 (100), 197 (22), 196 (26), 195 (21), 194 (22), 76 (22), 75 (23), 55 (48). Elemental Analysis for $C_{15}H_{16}BrNO$: calcd C, 58.84; H, 5.27; N, 4.57; found C, 58.71; H 5.23; N, 4.58.

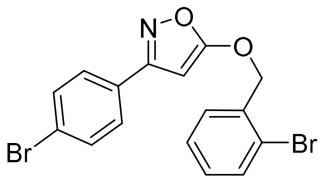

5-((2-bromobenzyl)oxy)-3-(4-bromophenyl)isoxazole 3c:

Compound **3c** was synthesized in 70 % yield using general procedure 1. M.p. 172-175 °C. ^{1}H NMR (acetone-d_6) : 7.88 (d, J = 8 Hz, 2H), 7.72 (d, J = 8 Hz, 2H), 7.64 (d, J = 4 Hz, 1H), 7.42 – 7.38 (m, 1H), 7.30 (d, J = 8 Hz, 1H), 7.11 (s, 1H), 6.99 – 6.97 (m, 1H), 5.46 (s, 2H). ^{13}C NMR :168.7, 161.4, 154.5, 133.4, 132.2, 129.0, 128.6, 128.2, 123.9, 123.1, 114.3, 111.8, 101.9, 61.9. GC-EIMS (m/z): 238 (88), 236 (88), 157 (97), 155 (89), 145 (94), 143 (100), 127 (63), 76 (72), 75 (77), 63 (84). Elemental Analysis for $C_{16}H_{11}Br_2NO_2$: calcd C, 46.98; H, 2.71; N, 3.42; found C, 47.03; H 2.68; N, 3.37.

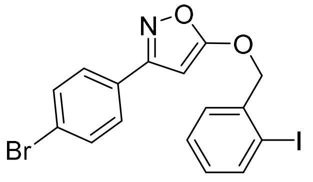

3-(4-bromophenyl)-5-((2-iodobenzyl)oxy)isoxazole 3d:

Compound **3d** was synthesized in 72 % yield using general procedure 1. M.p. 177-179. ^{1}H NMR (acetone-d_6) : 7.86 (t, J = 8 Hz, 3H), 7.71 (d, J = 8 Hz, 2H), 7.44 – 7.40 (m, 1H), 7.21 (d, J = 8 Hz, 1H), 7.10 (s, 1H), 6.86 – 6.83 (m, 1H), 5.44 (s, 2H). ^{13}C NMR :168.8, 161.4, 156.8, 140.0, 132.2, 129.8, 128.2, 123.9, 101.9, 85.92, 62.0. GC-EIMS (m/z): 238 (70), 236 (78), 219 (71), 157 (66), 155 (66), 92 (100), 76 (93), 75 (62), 64 (59), 63 (98). Elemental Analysis for $C_{16}H_{11}Br_2INO_2$: calcd C, 42.14; H, 2.43; N, 3.07;; found C, 43.01; H 2.53; N, 3.12.

Characterization data for compound 5a-f:

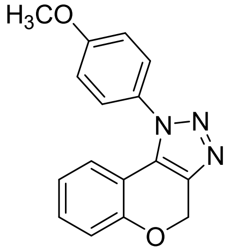

1-(4-methoxyphenyl)-1,4-dihydrochromeno[3,4-d][1,2,3]triazole 5a:

Compound **5a** was synthesized in 72 % yield using general procedure 2. M.p. 151-153 °C. ^{1}H NMR (chloroform-d) : 7.48 – 7.43 (m, 2H), 7.23 – 7.19 (m, 1H), 7.11 – 7.05 (m, 2H), 7.02 (d, J = 8 Hz, 1H), 6.90 (dd, J = 1.6, 7.6, 1H), 6.82 – 6.78 (m, 1H), 5.53 (s, 2H), 3.92 (s, 3H). ^{13}C NMR : 160.9, 153.8, 139.2, 130.7, 129.6, 128.4, 127.1, 122.4, 121.8, 117.9, 114.8, 114.0, 64.5, 55.7. GC-EIMS (m/z): 279 (18), 251 (35), 250 (38), 237 (17), 236 (100), 220 (16), 208 (31), 207 (33), 89 (15), 63 (17). Elemental Analysis for $C_{16}H_{13}N_3O_2$: calcd C, 68.81; H, 4.69; N, 15.05; found C, 68.85; H, 4.57; N, 15.12.

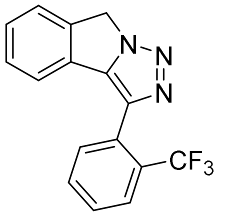

3-(2-(trifluoromethyl)phenyl)-8H-[1,2,3]triazolo[5,1-a]isoindole 5b:

Compound **5b** was synthesized in 72 % yield using general procedure 2. M.p. 128 – 130 °C. ^{1}H NMR (chloroform-d) : 7.87 (d, J = 1.2 Hz, 1H), 7.86 – 7.67 (m, 1H), 7.66 – 7.58 (m, 2H), 7.54 (d, J = 7.2 Hz, 1H), 7.42 – 7.36 (m, 3H), 5.42 (s, 2H). ^{13}C NMR :141.2, 140.9, 135.9, 132.6, 131.9, 129.9 (q, J_{C-F} = 30.7 Hz), 129.9 (q J_{C-F} = 2 Hz), 129.1, 128.8, 128.72, 127.9, 126.8 (q, J_{C-F} = 5 Hz), 124.3, 124.0 (q, J_{C-F} = 273 Hz), 121.6, 51.3. GC-EIMS (m/z): 239 (15), 212 (29), 198 (35), 157 (66), 197 (100), 184 (31), 178 (33), 169 (52), 151 (64), 134 (19), 107 (28). Elemental Analysis for $C_{16}H_{10}F_3NO_3$: calcd C, 63.79; H, 3.35; N, 13.95; found C, 64.01; H, 3.29; N, 14.04.

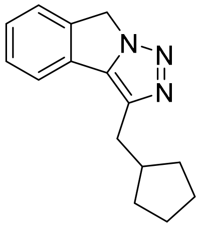

3-(cyclopentylmethyl)-8H-[1,2,3]triazolo[5,1-a]isoindole 5c:

Compound **5c** was synthesized in 72 % yield using general procedure 2. M.p. 107 - 109 °C. ^{1}H NMR (chloroform-d) : 7.62 (d, J = 8 Hz, 1H), 7.52 – 7.43 (m, 2H), 7.37 (td, J = 8, 12 Hz, 1H), 5.29 (s, 2H), 2.95 (d, J = 7 Hz, 2H), 2.41 – 2.30 (m, 1H), 1.85 – 1.74 (m, 2H), 1.70 – 1.62 (m, 2H) , 1.58 – 1.53 (m, 2H) , 1.34 – 1.25 (m, 2H). ^{13}C NMR : 140.8, 139.6, 138.9, 128.8, 128.7, 127.8, 124.3, 120.9, 51.0, 40.7, 32.5, 31.8, 25.1. GC-EIMS (m/z): 239 (15), 210 (63), 183 (33), 182 (100), 168 (28), 167 (17), 143 (36), 115 (18), 89 (21). Elemental Analysis for $C_{15}H_{17}N_3$: calcd C, 75.28; H, 7.16; N, 17.56; found C, 75.30; H, 7.11; N, 17.59.

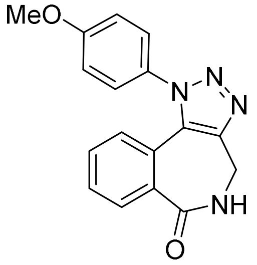

1-(4-methoxyphenyl)-4,5-dihydrobenzo[c][1,2,3]triazolo[4,5-e]azepin-6(1H)-one 5d:

Compound **5d** was synthesized in 72 % yield using general procedure 2. M.p. 180 – 183 °C. ^{1}H NMR (chloroform-d) : 8.15 (2, br 1H), 7.79 (dd, J = 1.2, 7.6 Hz, 1H), 7.65 (d, J = 9 Hz, 2H), 7.33 (t, J = 7.2 Hz, 1H), 7.05 – 7.02 (m, 3H), 6.76 (t, J = 7.6 Hz, 1H), 5.36 (s, 2H), 3.87 (s, 3H). ^{13}C NMR :167.6, 159.8, 134.0, 131.9, 131.7, 131.4, 128.9, 128.8, 128.6, 127.3, 122.5, 114.9, 55.6, 35.7. GC-EIMS (m/z):306 (48), 304 (72), 282 (100), 247 (25), 207 (20), 185 (64), 149 (32), 127 (67). Elemental Analysis for $C_{17}H_{14}N_4O_2$: calcd C, 66.66; H, 4.61; N, 18.29; found C, 66.58; H, 4.64; N, 18.22.

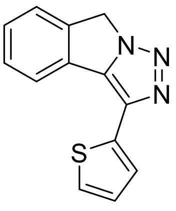

3-(thiophen-2-yl)-8H-[1,2,3]triazolo[5,1-a]isoindole 5e:

Compound **5e** was synthesized in 72 % yield using general procedure 2. M.p. 178 – 181 °C. ^{1}H NMR (chloroform-d) : 7.86 (d, J = 8 Hz, 1H), 7.74 (dd, J = 2.8, 1.2 Hz, 1H), 7.64 (dd, J = 4.8, 1.2 Hz, 1H) 7.54 – 7.50 (m, 1H), 7.48 – 7.43 (m, 2H), 7.40 (m, 1H), 5.32 (s, 2H); ^{13}C NMR : 140.8, 138.2, 134.8, 132.0, 128.6, 128.1, 127.8, 126.5, 126.3, 124.1, 122.3, 121.1, 58.4. GC-EIMS (m/z): 239 (15), 211 (87), 210 (100), 184 (32), 166 (19), 139 (34), 102 (17), 92 (58), 79 (22), 63 (27). Elemental Analysis for $C_{13}H_9N_3S$: calcd C, 65.25; H, 3.79; N, 17.56; S, 13.40; found C, C, 65.21; H, 3.81; N, 17.51; S, 13.46.

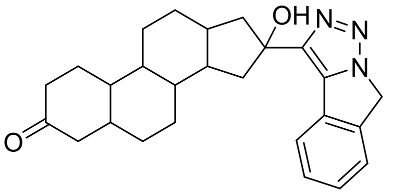

16-(8H-[1,2,3]triazolo[5,1-a]isoindol-3-yl)-16-hydroxyhexadecahydro-3H-cyclopenta[a]phenanthren-3-one 5f:

Compound **5f** was synthesized in 72 % yield using general procedure 2. M.p. 188 - 190 °C. ^{1}H NMR (chloroform-d) : 7.99 (d, J = 5 Hz, 1H), 7.48 – 7.38 (m, 3H), 5.78 (s, 1H), 5.32 (s, 2H), 2.48 – 2.45 (m, 1 H), 2.29 – 2.04 (m, 7 H), 1.91 – 1.89 (m, 1H), 1.62 – 1.58 (m, 4H), 1.41 – 1.40 (m, 2H), 1.25 – 1.17 (m, 9H). ^{13}C NMR : 200.0, 166.9, 144.4, 141.2, 141.1, 128.9, 128.8, 128.2, 124.7, 123.9, 83.5, 48.9, 48.3, 47.9, 42.6, 41.2, 38.2, 36.7, 35.7, 32.5, 30.8, 29.8, 26.6, 26.2, 24.5, 14.5. GC-EIMS (m/z): 417 (28), 401 (16), 403 (19), 313 (22), 312 (17), 298 (67), 272 (100), 246 (47), 208 (24), 198 (32). Elemental Analysis for $C_{26}H_{31}N_3O_2$: calcd C, 74.79; H, 7.48; N, 10.06; found C, 74.71; H, 7.52; N, 10.12.

E-factor for synthesis of compound 5a-5f (1 mmol scale)

Assuming that the reaction medium and the catalyst can be recycled:

5a: {[49 mg ($MesCO_2H$) + 276 mg (K_2CO_3) + 1 g (water used to wash the solid) + 407 mg (starting material)] – [242 mg (isolated product)]}/[242 mg (product)] = **6.1**

5b: {[49 mg ($MesCO_2H$) + 276 mg (K_2CO_3) + 1 g (water used to wash the solid) + 429 mg (starting material)] – 252 mg (isolated product)}/[252 mg (product)] = **5.9**

5c: {[49 mg ($MesCO_2H$) + 276 mg (K_2CO_3) + 1 g (water used to wash the solid) + 367 mg (starting materials)] – 186 mg (isolated product)}/[186 mg (product)] = **8.1**

5d: {[49 mg ($MesCO_2H$) + 276 mg (K_2CO_3) + 1 g (water used to wash the solid) + 434 mg (starting materials)] – 208 mg (isolated product)}/[208 mg (product)] = **7.4**

5e: {[49 mg ($MesCO_2H$) + 276 mg (K_2CO_3) + 1 g (water used to wash the solid) + 367 mg (starting materials)] – 201 mg (isolated product)}/[201 mg (product)] = **7.4**

5f: {[49 mg ($MesCO_2H$) + 276 mg (K_2CO_3) + 1 g (water used to wash the solid) + 1g (Polarclean) + 11 mg (catalyst) + 545 mg (starting materials)] – 217 mg (isolated product)}/[217 mg (product)] = **12.4**

Calculation of E-factor for representative literature procedure

The procedures and E-factor calculations described below are representative. In the evaluation of the results obtained, it should be considered that these procedures have not been specifically optimized for waste minimization protocols.

Reference	E-factor Calculation
Ackermann et al., 2010. Org Lett., 12, 2056 – 2059	(0.96 mmol, yield 96%)=[(0.287(triazole) + 0.011 g ($Pd(OAc)_2$) + 0.200 g ($Cu(OAc)_2$) + 0.500 g (PivOH) + 2.61 g (toluene, density = 0.87 g/mL) + 53 g (NH_4Cl density = 1.06 g/mL) + 135 g (EtOAc density = 0.902 g/mL) + 150 g (water)] – [0.274 g (isolated product)] / 0.274 g (isolated product) = **1245** (column chromatography was not considered in *E*-factor calculation)
Chen et al., 2013. RSC Adv. 3, 2710 – 2719	(0.84 mmol, yield 84%)=[(0.377 g (triazole) + 0.005 g ($Pd(OAc)_2$) + 0.967 g (TBAI) + 9.4 g (DMF, density = 0.944 g/mL) + 360 g (EtOAc density = 0.944 g/mL) + 400 g (water) + 238 g (brine density = 1.19 g/mL)] – [(0.209 g (isolated product))] / 0.209 g (isolated product) = **4825** (column chromatography was not considered in *E*-factor calculation)
Nagarjuna Reddy et al., 2012. Eur. J. Org. Chem. 2013–2022.	(0.35 mmol, yield 70 %)=[(0.009 g (CuI) + 0.001 g (TMEDA) + 0.944 g (DMF, density = 0.944 g/mL) + 0.066 g (O-Alkynyliodophenol) + 0.074 g (azide) + 0.120 g (Lithium tert butoxide) + 10.82 g (EtOAc density = 0.944 g/mL) + 10 g (water) + 12 g (brine density = 1.19 g/mL)) – (0.097 g (isolated product))] / 0.097 g (isolated product) = **349** (column chromatography was not considered in *E*-factor calculation)

General procedure for the recycling of the catalyst and the reaction medium in the intramolecular C–H activation of triazoles.

All reactions were run on a 2 mmol scale, using 2 mL of pure Polarclean as reaction medium. Triazole (2 mmol, 1 eq), MesCO2H (0.6 mmol, 0.3 eq), K2CO3 (4 mmol, 2 eq), Pd(OAc)2 (0.1 mmol, 0.05 eq) were charged in a screw capped vial equipped with a magnetic stirring bar and the recycled liquid phase obtained from the previous reaction run was added. The reaction was heated at 120 °C under stirring for 24h. The product was filtered with a Büchner funnel, washed with water (2 mL) and dried under vacuum.
